# Retrospective analysis of seasonal patterns in atrial fibrillation incidence and temperature fluctuations

**DOI:** 10.6026/973206300214293

**Published:** 2025-12-15

**Authors:** Pooja Saithya Pillarisetti, Karthik Mathiyalagan, Shanmukha Koppolu, Sorabh Sharma, Markondapatnaikuni Navya, Adithya Chandran

**Affiliations:** 1 Department of General Medicine, A.C.S.R Government Medical College Nellore Andhra Pradesh, India; 2 Department of Trauma Surgery, King's College Hospital, London, United Kingdom; 3 Department of Trauma and Orthopaedics, Barts NHS Trust, London, England, United Kingdom; 4 Department of Internal Medicine, University of Arizona, Arizona, USA; 5 Department of Emergency Medicine, Maharajah's Institute of Medical Sciences, Nellimarla, Vizianagaram andhra Pradesh, India; 6 Department of Internal Medicine, Indira Gandhi Medical College and Research Institute, Pondicherry, India

**Keywords:** Atrial fibrillation, seasonality, temperature, cardiovascular risk, retrospective analysis, winter onset

## Abstract

Atrial fibrillation (AF) remains the most common sustained arrhythmia contributing significantly to global morbidity and mortality. This retrospective study analyzed 126,389 adult patients across three tertiary hospitals from 2018 to 2022 to assess the seasonal variation in AF incidence with respect to ambient temperature. The incidence of AF was significantly higher during winter and early spring, showing an inverse correlation with mean daily temperature (r = -0.81, p < 0.001), particularly among elderly, hypertensive, and diabetic individuals. Cold-induced vasoconstriction, increased sympathetic tone, and inflammatory responses may underlie the seasonal rise in AF admissions. Recognizing these temperature-related variations can assist in identifying high-risk periods and guide preventive strategies to mitigate the burden of AF.

## Background:

Atrial fibrillation (AF) is the most common sustained cardiac arrhythmia in the world, resulting in significant morbidity and mortality, including an increased risk of stroke, heart failure and cardiovascular mortality [1]. The incidence of AF increases with age and is associated with well-established risk factors such as hypertension, obesity, diabetes mellitus, valvular heart disease and structural heart abnormalities [2]. Although there have been improvements in both pharmacological and interventional therapies for AF, it continues to impose a significant clinical and economic burden on health systems worldwide [3]. Recent evidence also suggests that environmental and seasonal factors are associated with the initiation and worsening of AF, in addition to conventional risk factors [4]. Seasonal trends are documented in cardiovascular-related morbidity and mortality, with winter seasons often associated with peaks of arrests [5]. Temperature changes, especially, cold exposure, have been suggested as triggers for AF through various physiologic mechanisms. Cold exposure can elicit peripheral vasoconstriction, elevate systemic blood pressure, increase sympathetic nervous system activation and induce inflammatory signaling, all of which may lead to increased myocardial stress and a greater risk of atrial arrhythmogenesis [6]. Alternatively, very hot weather can lead to cardiovascular stress due to dehydration, electrolyte disturbances and increased heart rates. Epidemiological investigations on the relationship between seasonal or temperature variation have reported inconsistent associations with AF risk [7]. Some retrospective studies have shown a higher rate of AF in winter months while others have shown little or no seasonal association [8]. That said, differences in findings may be related to variability in study populations, geographic location, climatic conditions and data collection. To date, very few studies have systematically assessed the associations between daily ambient temperatures and AF events on a population basis, which has created further gaps in the knowledge [9]. Understanding the impact of seasonal and environmental variations on AF is clinically important for many reasons. It may identify clinicians' periods of increased risk for arrhythmia onset, enabling targeted monitoring, early intervention and modification of preventive strategies, particularly in older adults and those with other multiple comorbidities [10]. Additionally, knowledge of environmental triggers may yield public health recommendations about specific management of indoor temperature, adopting lifestyle modifications during extreme weather and the planning of resources for emergent cardiovascular care during periods of increased risk [11]. In light of the clinical importance and existing knowledge gaps, there is a need for additional exploration of seasonal trends and temperature changes for the incidence of AF with large-scale retrospective studies which can provide further insights into temporal patterns inform risk stratification and improve prevention strategies for atrial fibrillation across a variety of populations [12]. Therefore, it is of interest to report the seasonal patterns and temperature-related variations in atrial fibrillation incidence to inform preventive strategies and optimize patient outcomes.

## Materials and Methods:

This retrospective observational study analyzed electronic health records from three tertiary care hospitals over a 5-year period (2018-2022). A total of 126,389 adult patients (≥18 years) were included after excluding those with pre-existing chronic AF, pacemakers, or incomplete meteorological linkage. Daily hospital admissions with a new diagnosis of atrial fibrillation (ICD-10 code I48.0-I48.91) were recorded. Meteorological data, including daily average temperature (°C), humidity and barometric pressure, were obtained from official regional meteorological stations and synchronized with each admission date. Seasonal periods were categorized as winter (December-February), spring (March-May), summer (June-August) and Autumn (September-November). Data were stratified by age, sex, comorbidities (hypertension, diabetes, coronary artery disease) and location. Statistical analysis was performed using R (version 4.2.2). Incidence rates were calculated per 100,000 population/month. Chi-square tests and ANOVA were used to assess seasonal differences. Pearson correlation coefficients determined the relationship between temperature and AF incidence. A p-value < 0.05 was considered statistically significant.

## Results:

In an investigation of 126,389 patient records, certain patterns of seasonality were evident during the incidence of atrial fibrillation (AF) whereby there was a peak of AF admission in winter and early spring. AF incidence was shown to be less frequent during colder daily average temperatures, particularly among the elderly population (aged ≥65 years) and people with pre-existing cardiovascular risk factors, the latter having the greatest effect. The findings suggest that there is an increase in AF among the elderly population and with hypertensive and diabetic patients. Although the number of days patients experienced AF was modestly higher in males than females, males and females demonstrated the same seasonal incidence. Hospital admissions for AF, as well as lengths of stay (LOS), were greater among patients, particularly, both with lower temperature and higher barometric pressure, likely due to the burden of comorbidity. The findings indicate environmental and patient factors contribute to the incidence, seasonality and burden of AF. Table 1 (see PDF) shows that AF incidence was highest during winter months and lowest in summer, indicating significant seasonal variation. Table 2 (see PDF) shows that winter had significantly higher mean monthly AF incidence compared to spring, summer and autumn. Table 3 (see PDF) shows that elderly patients (≥65 years) exhibited the sharpest seasonal variation in AF occurrence compared to younger age groups. Table 4 (see PDF) shows that males had slightly higher AF incidence overall, but both sexes followed a similar seasonal trend. Table 5 (see PDF) shows a strong negative correlation between mean daily temperature and AF incidence, indicating higher AF rates on colder days. Table 6 (see PDF) shows those days with temperatures below 10°C had significantly higher AF admissions compared to warmer days. Table 7 (see PDF) shows that hypertensive individuals exhibited greater seasonal sensitivity in AF onset compared to non-hypertensive patients. Table 8 (see PDF) shows that diabetic patients also displayed seasonal amplification in AF incidence, similar to hypertensive individuals. Table 9 (see PDF) shows that AF admissions were more frequent on days with both low temperature (<10°C) and high barometric pressure (>1020 hPa). Table 10 (see PDF) shows that hospital length of stay for AF patients was longer during winter, likely due to increased comorbid decompensations.

## Discussion:

This retrospective analysis revealed a clear seasonal trend in atrial fibrillation (AF) incidence, with a consistent peak during winter and lowest rates during summer. The inverse correlation between ambient temperature and AF onset supports the hypothesis that colder weather exacerbates cardiovascular stress through increased sympathetic activation, peripheral vasoconstriction and inflammatory responses [13]. These effects may act as acute triggers for AF, especially in individuals with pre-existing comorbidities such as hypertension, diabetes, or advanced age. The elderly population showed the most pronounced seasonal variation, likely due to reduced autonomic flexibility and impaired thermoregulatory capacity. Moreover, hypertensive and diabetic patients were particularly sensitive to temperature-induced AF variation, indicating a compounded risk from both intrinsic and environmental factors [14]. These findings align with existing literature but are strengthened by the large sample size and comprehensive climate linkage. Regional disparities, especially between northern and southern zones, highlight the influence of geographic climate on cardiac rhythm disorders [15]. Additionally, longer hospital stays and higher complication rates during colder months may reflect both greater severity of AF episodes and overlapping winter-related respiratory or cardiovascular illnesses. The observed patterns suggest that proactive monitoring, medication adjustments and thermal protection strategies during colder seasons may be critical in AF management [16]. This study emphasizes the need for clinicians and public health systems to integrate seasonal risk awareness into preventive care, particularly in high-risk groups and colder regions.

## Conclusion:

Atrial fibrillation incidence shows a clear seasonal pattern, peaking during colder months and correlating inversely with ambient temperature. Elderly individuals and those with comorbidities are especially vulnerable to cold-induced AF episodes. Seasonal awareness and preventive strategies could reduce AF burden and improve outcomes in at-risk populations.
